# Unveiling the dynamics and therapeutic potential of m^6^A methyltransferases and demethylases in liver diseases

**DOI:** 10.7150/ijbs.120058

**Published:** 2025-10-01

**Authors:** Ya-Ning Chen, Sai Zhu, Li-Jiao Sun, Rong-Rong Zhou, Rui Zheng, Xiao-Feng Li, Liang-Yun Li, Si-Jin Sun, Yu-Xin Zhao, Cheng Huang, Xiao-Ming Meng, Lei Zhang, Xiong-Wen Lv, Hua Wang, Xin Chen, Jun Li

**Affiliations:** 1 Inflammation and Immune Mediated Diseases Laboratory of Anhui Province, Anhui Institute of Innovative Drugs, The Third Affiliated Hospital, School of Pharmacy, Anhui Medical University, Hefei 230032, China.; 2 The Key Laboratory of Anti-inflammatory and Immune Medicines, Anhui Medical University, Ministry of Education, Hefei 230032, China.; 3 Institute for Liver Diseases of Anhui Medical University, ILD-AMU, Anhui Medical University, Hefei 230032, China.; 4 Department of Pharmacology and Laboratory of Cerebrovascular Pharmacology, School of Pharmaceutical Sciences, Soochow University, Suzhou 215123, China.

**Keywords:** m6A modification, liver diseases, methyltransferase, demethylase

## Abstract

N^6^-methyladenosine (m^6^A), a well-known adenosine modification with newly recognized epigenetic functions, reportedly participates in the development of diverse liver diseases. Methyltransferases and demethylases, commonly referred to as “writers” and “erasers”, respectively, play crucial roles in maintaining the balance of m^6^A modification. In liver disease research specifically, the functioning of these enzymes has piqued significant interest, revealing new perspectives on molecular pathogenic mechanisms. Writer proteins collaborate with co-factors to install m^6^A modification on RNA, while eraser proteins, exemplified by Fto and Alkbh5, remove modifications via different mechanisms. In liver diseases, the two are not simply antagonistic, but rather act jointly to affect disease progression. By focusing this review on the mechanisms of methyltransferases and demethylases in various liver diseases, we seek to enhance comprehension of m^6^A modification's role and support the advancement of related research and treatment strategies.

## 1. Introduction

Since the landmark discovery of pseudouridine as the first chemically modified nucleoside in the 1950s^1^, ^2^. In recent years, RNA modification biology has attracted renewed widespread attention due to the recognition of the prevalence and functional significance of internal mRNA modifications, particularly N^6^-methyladenosine (m^6^A)^3^. Spurred by advances in high sensitivity, high throughput sequencing methodologies for transcriptome wide mapping, N^6^-methyladenine modifications have now been documented across phylogenetically diverse organisms spanning prokaryotes to humans^4^. Among these modifications, m^6^A stands out due to its exceptional abundance and recognition as a pivotal post-transcriptional regulator^5^. This modification serves as an important regulatory marker in multiple RNA species, including mRNA, tRNA, rRNA, circRNA, miRNA, and lncRN^1^, ^6^.

Spatiotemporally coordinated interactions among “writers” “erasers” and “readers” triads govern the m^6^A epitranscriptome. The “writers” complex spearheaded by Mettl3, Mettl14, and Mettl16 catalytic triumvirate within the Mettl methyltransferase family collaborates with “erasers” to establish reversible modification landscapes^7^. As shown in (Table 1), key contributions to the m^6^A methyltransferase complex include the following: Mettl3 interacts with S-adenosylmethionine (SAM) to achieve methyl transfer^3^ and forms heterodimers with Mettl14 to enhance catalytic efficiency and promote substrate binding^8^; Mettl16 is involved in specific RNA modifications; Wilms tumor 1-associating protein (Wtap) assists in the localization and catalysis of Mettl3/Mettl14^9^; RNA-binding motif protein Rbm15 and Rbm15b recruits the complex to specific RNA sites^10^; Casitas B-lineage lymphoma transforming sequence-like 1 (Cbll1, also called E3 ubiquitin-protein ligase Hakai) maintains the stability of modifications^11^; Vir-like m^6^A methyltransferase associated (Virma), also designated as Kiaa1429, guides regional-specific methylation^12^; and zinc finger CCCH-type containing 13 (Zc3h13, also known as Flacc) maintains the nuclear localization of the related complex^13^.

Among eraser proteins, fat mass and obesity-associated protein (Fto) and AlkB homolog 5 (Alkbh5) demonstrate particularly distinctive characteristics^14^. Fto, the first demethylase to be discovered, exhibits different substrate preferences in the nucleus and cytoplasm and removes methyl groups via oxidation reaction^15^, ^16^. Alkbh5 is an endogenous demethylase that mainly mediates the demethylation of the 3'-untranslated region (UTR) of specific transcripts^17^, ^18^.

The impact of m^6^A modification on gene expression also requires the involvement of reader proteins, particularly members of the insulin-like growth factor 2 mRNA-binding protein (Igf2bp) and YTH domain family^19^, ^20^. Reader proteins, by interacting with other molecules, decipher the information carried by m^6^A modifications to regulate the metabolic processes of mRNAs, such as splicing, nuclear export, translocation, and stability^21^. Critically, disruption of the dynamic equilibrium between m^6^A methyltransferases and demethylases induces reader protein dysregulation, thereby driving disease pathogenesis. This mechanistic cascade has been validated by the following studies: The opposing actions of Mettl14 mediated m^6^A methylation and Alkbh5-dependent demethylation on Tgf-β1 mRNA establish a dynamic regulatory switch that differentially controls hepatic stellate cell activation and profibrotic signaling^22^, ^23^. Alcoholic hepatitis exhibits Mettl3-mediated m^6^A hypermethylation and Fto deficient demethylation of Il-17r mRNA, driving its pathological overexpression and inflammation amplification^24^.

Recognizing the critical role of m6A modification in liver diseases, our group has systematically investigated m^6^A-related proteins. We demonstrated how m^6^A readers regulate gene expression and disease progression^25^, highlighted m^6^A's importance in liver pathophysiology^26^, and revealed that Wtap mediates m^6^A modification of circDcbld2 and interacts with Igf2bp2, uncovering a key mechanism in hepatic fibrosis^27^. However, comprehensive reviews elucidating the dynamic regulatory mechanisms of m^6^A methyltransferases and demethylases in hepatic disorders remain notably lacking.

This review systematically synthesizes recent advances in m^6^A modification patterns across major liver diseases, including acute liver injury (ALI), viral hepatitis, nonalcoholic fatty liver disease(NAFLD), hepatic fibrosis (HF), and hepatocellular carcinoma (HCC), with the ultimate goal of developing mechanism based therapeutic strategies to improve clinical outcomes.

## 2. Formation and removal of THE m^6^A modification

**Figure 1:**
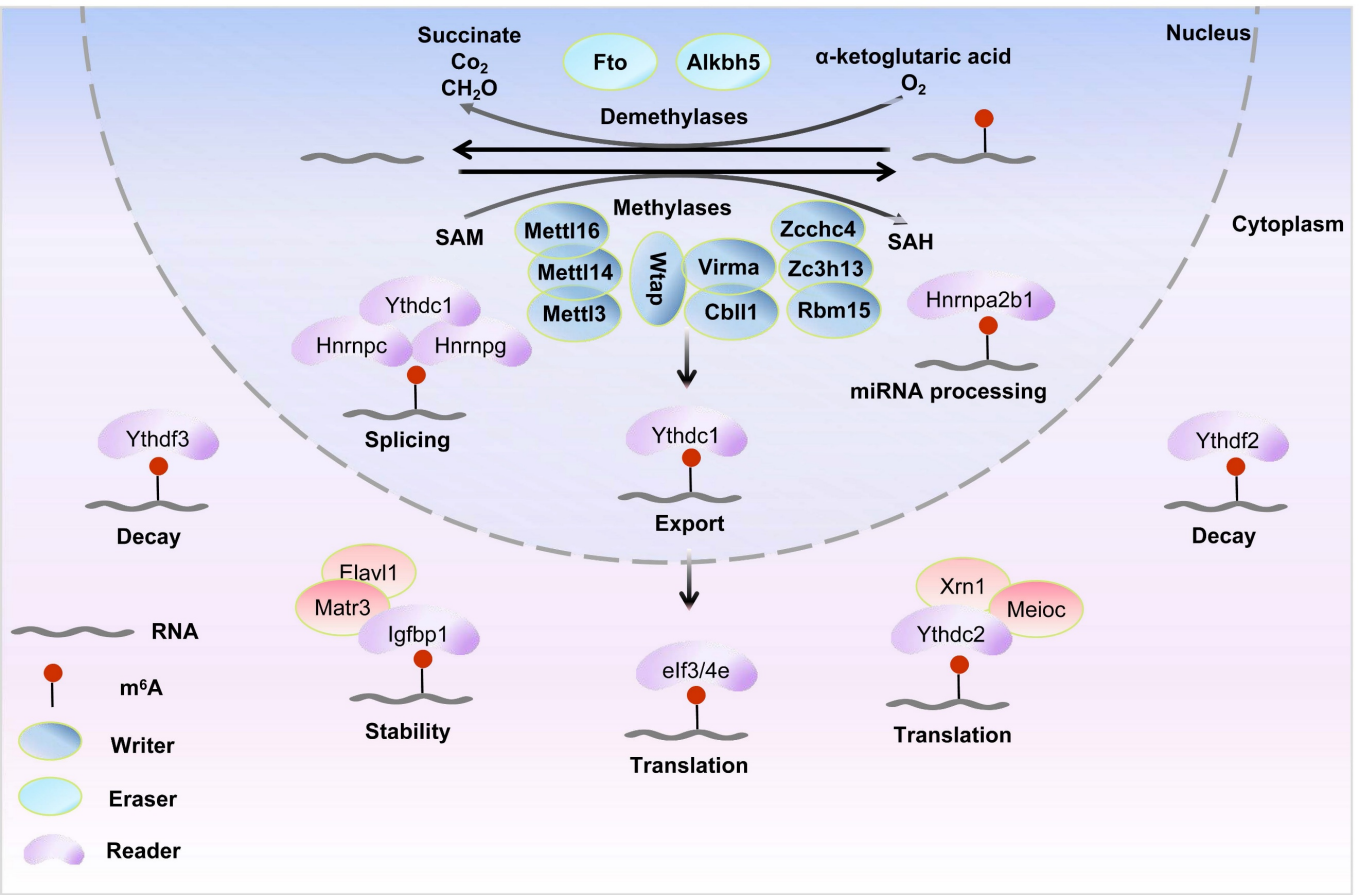
** Reversible m^6^A modification and molecular functions on mRNA.** The m^6^A methylation is catalyzed by the writer complex including Mettl3, Mettl14, Mettl16, Wtap, Virma, Rbm15/15b, Cbll1, Kiaa1429, and Zc3h13. The m^6^A modification is erased by demethylases including Fto and Alkbh5. Methylases and demethylases achieve the reversible regulation of m^6^A modification via dynamic equilibrium.

As shown in (Figure 1), the m^6^A modification, which is the most common internal epigenetic mark in mRNA, is added by m^6^A methyltransferases or writers and is removed by demethylases or erasers (e.g., Fto and Alkbh5). These modifications can be recognized by m^6^A reader proteins, thereby participating in the post-transcriptional regulation of RNA^28^.

### 2.1 m^6^A methyltransferases

m^6^A modification, catalyzed by specific methyltransferases, plays extensive roles in regulating gene expression and RNA metabolism. Precise control of this modification is crucial for maintaining its homeostasis, and in-depth investigation of these enzymes will facilitate the elucidation of RNA epigenetic regulatory mechanisms.

#### 2.1.1 m^6^A methyltransferases of the Mettl family

The Mettl family proteins (including Mettl3, Mettl14, Mettl5, and Mettl16) constitute the core catalytic machinery for m^6^A deposition^29^. As introduced in Section 1, Mettl3 serves as the catalytic subunit that directly binds SAM to transfer methyl groups to RNA substrates, while Mettl14 acts as an allosteric activator enhancing Mettl3's RNA-binding affinity and catalytic efficiency within the heterodimeric complex^30^. Structural studies reveal that the catalytic site of Mettl3 is the exclusive domain for SAM binding, confirming its role as the sole active center^31^, ^32^. Furthermore, Mettl14's intrinsically disordered C-terminal domain interacts with protein arginine methyltransferase 1 (Prmt1) to maintain complex integrity and promote RNA substrate engagement, thereby augmenting methylation activity^33^, ^34^. Notably, beyond its catalytic function, Mettl3 contains a CCCH-type zinc-binding motif essential for in vitro RNA methylation^32^. Mettl16 regulates Mat2a mRNA splicing via methylation of its hairpin structure and modulates U6 snRNA methylation to influence mRNA splicing^35^, while Mettl5, stabilized by dimerization with Trmt112, mediates m^6^A modification at position A1832 of 18S rRNA^36^, ^37^.

To ensure proper m^6^A modification, Mettl3 and Mettl14 bind to Wtap, forming the Mettl3/Mettl14/Wtap complex identified via arabidopsis screening^38^. Wtap is essential for directing Mettl3/Mettl14 to nuclear speckles and boosting their catalytic activity *in vivo*^39^, ^40^. The complex primarily methylates the 3'-UTR of mRNA near stop codons within the DRACH motif (D=A/U/G, R=A/G, H=A/C/U), regulating mRNA processes^41^.

#### 2.1.2 Other m^6^A methyltransferases

In the realm of m^6^A modification, other pivotal factors and proteins are also intricately involved in the m^6^A methyltransferase machinery and its regulatory network. Virma binds RNA-dependently to polyadenylation factors, including cleavage and polyadenylation specificity factor subunits Cpsf5 and Cpsf6^42^, and recruits the Mettl3-Mettl14-Wtap methyltransferase complex to catalyze site-specific m^6^A methylation proximal to mRNA stop codons and within 3'-UTRs^43^. The Virma-Wtap complex enhances the catalytic activity of Mettl3-Mettl14 toward target RNAs, thereby modulating cellular m^6^A modification levels^44^. This complex recruits the methyltransferase machinery to DRACH motif-enriched RNA regions through its interaction with homologous RNA-binding proteins Rbm15 and Rbm15b^45^, ^46^. In *Drosophila* models, Hakai/Cbll1 mutants exhibit >50% reduction in mRNA m^6^A, highlighting its role in maintaining modification homeostasis^47^. Mechanistically, Hakai/Cbll1 interacts with Mettl3, Mettl16, and Virma via its E3 ubiquitin ligase activity to stabilize m^6^A modifications^48^. In mouse embryonic stem cells, Zc3h13 depletion reduces m^6^A levels and mislocalizes the Wtap-Virma-Hakai/Cbll1 complex to the cytoplasm, demonstrating its essential role in maintaining nuclear compartmentalization of the m^6^A methyltransferase machinery^49^. Additionally, Zc3h13 bridges the Rbm15-Wtap-Fl(2)d complex to the mRNA-binding factor Nito, facilitating efficient m^6^A deposition^50^. Zcchc4, as a recently reported potential RNA methyltransferase^51^, is conserved in other multicellular model organisms, but absent in yeast. Structural analysis of Zcchc4 revealed a putative m^6^A methyltransferase domain with a conserved catalytic DPPF motif and a CCHC-ZNF domain. These two domains cooperate to ensure Zcchc4 effectively mediates m^6^A modification at the A4220 site of 28S rRNA^52^, ^53^.

In summary, Mettl-family methyltransferases and their associated constitute the core machinery for m^6^A methylation. Their precisely regulated protein interactions dynamically modulate mRNA m^6^A levels, and mechanistic insights into this network provide critical insights into liver pathophysiology.

### 2.2 m^6^A demethylases

The m^6^A modification can be reversed via active demethylation by the m^6^A demethylases Fto or Alkbh5, illustrating that methylation-dependent processes are reversible and controllable. As the first RNA demethylase to be identified, Fto is demonstrably capable of catalyzing the removal of methyl groups from RNA^54^, ^55^. In contrast to Fto, Alkbh5 was identified through biochemical screening^56^. Fto and Alkbh5 exhibit distinct characteristics and functions during m^6^A modification, which may result in different regulatory patterns and biological effects.

#### 2.2.1 Context-dependent RNA demethylation by Fto

Fto, belonging to the non-heme Fe(II)/α-ketoglutarate-dependent AlkB dioxygenase family, exhibits functional similarity with Abh1-3 (oxidative demethylation of N-methylated DNA/RNA bases) and Abh8 (hydroxylation of tRNA wobble uridine)^57^-^59^. Experimental evidence have demonstrated that Fto can demethylate m³T and m³U in single-stranded (ss) DNA and ssRNA *in vitro*^60^, but its activity is lower than those of other family proteins. The latest crystal structure reveals that Fto prefers single-stranded nucleic acids (ssNAs) as substrates^61^, suggesting that its function may be modulated by context-dependent factors such as substrate conformation, reflecting the dynamic distribution of ssNAs across cellular compartments and physiological or pathological conditions. In mRNA, where m^6^A is the most abundant modification (3-5 marks per transcript)^62^. Research findings indicate that Fto targets m^6^A in mRNA, N^6^,2'-O-dimethyladenosine (m^6^Am) at the 5'-cap, and m^1^A in tRNA, thereby regulating these RNA modifications^63^, ^64^. Given that mRNA exists in a dynamic state shaped by nuclear cytoplasmic transport and protein interactions, the accessibility of m^6^A to Fto is likely context-dependent^65^. Given that Fto's activity on substrates can be influenced by environmental factors, it is reasonable to infer that its effect on m^6^A might also be context dependent.

Indeed, Fto exhibits compartment specific substrate preferences, underscoring its environmental sensitivity^66^. In the nucleus, where m^6^A is abundant, Fto functions as an Fe(II)/α-ketoglutarate-dependent dioxygenase to demethylate m^6^A via oxidative hydroxylation, producing formic acid/methanol and restoring adenosine^67^. Conversely, in the cytoplasm, Fto preferentially targets 5'-cap m^6^Am, potentially regulating mRNA stability, translation efficiency, and decay pathways^68^. These differential actions in the nucleus and cytoplasm clearly illustrate how the cellular environment shapes the substrate selection and catalytic function of Fto.

#### 2.2.2 Substrate and roles of Alkbh5

Alkbh5, the second identified RNA demethylase, has only one known substrate: m^6^A^56^. As one of nine members of the AlkB family of ferrous iron- and 2-oxoglutarate-dependent nucleic acid oxygenases (Naoxs), it reportedly catalyzes the demethylation of m^6^A in RNA. This finding has improved our understanding of its substrate recognition specificity^63^. Crystallographic studies reveal that Alkbh5, primarily mediates the demethylation of m^6^A in the 3'-UTR of specific transcripts^69^. The expression patterns of Alkbh5 and Fto exhibit significant across tissues. For instance, Alkbh5 is most highly expressed in the testes, while Fto is predominantly expressed in the brain^70^. This differential tissue expression may be one explanation for the distinct biochemical pathways through which these enzymes participate in m^6^A demethylation. Comprehensive characterization of Alkbh5's properties, functions, and tissue specificity is crucial for mechanistically understanding its regulation of RNA methylation in biological processes.

## 3. Biological function and interaction of methyltransferaseS and demethylaseS

In the field of epigenetics, m^6^A modification is one of the most prevalent and well-studied RNA modifications, demonstrating widespread involvement in all aspects of mRNA metabolism, including export, translation, stability, and splicing. Specifically, methyltransferases are responsible for adding methyl groups to mRNA to complete the writing, demethylases remove these methyl groups to achieve erasure, and readers recognize the m^6^A modification sites on mRNA.

### 3.1 Regulation of mRNA splicing

The maturation of pre-mRNA involves 5'-capping, 3'-polyadenylation, and splicing^71^. Writer proteins like Mettl3 mediate m^6^A methylation at specific sites on introns and exons, correlating with transcription start sites (TSS) and stop codons to regulate RNA stability and splicing^72^, ^73^. While m^6^A does not disrupt Watson-Crick base pairing, it reduces double-stranded RNA stability by 1.4 kcal/mol^74^ while stabilizing surrounding structures^75^ or promoting the folding of adjacent RNA sequences^76^, thereby influencing spliceosome assembly^77^. These modifications can be recognized by readers such as Hnrnpg, which further regulate mRNA splicing and stability. Ythdc1 (also known as Dc1) is another important reader protein. Some studies have shown that Ythdc1 interacts with splicing regulators, including Src-associated in mitosis, 68kDa (Sam68)^78^, splicing factor Sc35^79^, and serine-arginine-rich splicing factors Srsf1 and Srsf3^80^, suggesting its involvement in splicing regulation. However, others reports indicate that Ythdc1 selectively regulates mRNA splicing by promoting the binding of Srsf3 while inhibiting the activity of Srsf10^81^. Fto-mediated m^6^A demethylation reduces Rbm15 binding at splicing junctions (e.g., 5'-AG|GUAAGU/3'-CAG|G), disrupting recruitment of Srsf/Hnrnp family proteins and increasing exon skipping in prostate cancer^82^. Collectively, writers and erasers coordinately regulate RNA splicing via dynamic m^6^A modification, with their dysregulation linked to disease pathogenesis.

### 3.2 Regulation of mRNA nuclear export

In eukaryotes, mRNA nuclear export critical for cytoplasmic protein synthesis depends on dynamic regulation by m^6^A writer and eraser proteins. The Mettl3-Mettl14 heterodimer, forming the core of the writer complex with Wtap and Virma, establishes context-specific m^6^A methylation on pre-mRNA^83^. This complex deposits m^6^A modifications to with site and transcript-specific patterns. For example, in human embryonic stem cells, Smad2/3 transcription factors bind to Mettl3, Mettl14, and Wtap, promoting m^6^A modification of target transcripts^84^. These m^6^A marks act as "identity tags," altering mRNA secondary structure to modulate interactions with nuclear export machinery components, thereby influencing export efficiency.

Fto and Alkbh5, as key m^6^A erasers, collaborate with writer proteins to maintain dynamic m^6^A homeostasis and regulate mRNA nuclear export. While Fto's demethylation activity toward m^6^A remains controversial, it demonstrates strong catalytic efficiency toward m^6^Am, primarily influencing snRNA methylation^85^. In contrast, nuclear localized Alkbh5 is a validated m^6^A demethylase. In cancer cells, Alkbh5 upregulation reduces m^6^A modification on specific mRNAs (e.g., Nanog and Foxm1), modifying their biophysical properties and potentially impairing nuclear export^86^, ^87^. This reduction in m^6^A modification alters the properties of these mRNAs and may consequently affect their nuclear export.

Reader proteins facilitate mRNA nuclear export by directly or indirectly mediating m^6^A-modified mRNA interactions. The nuclear reader Ythdc1 binds m^6^A-modified mRNAs and recruits Srsf3^80^, a critical adaptor in the nuclear export factor (Nxf)1-dependent export pathway, to drive nuclear export^88^. Another reader protein, the shuttling reader Fmrp (fragile X mental retardation protein) preferentially associates with m^6^A modified transcripts, collaborating with chromosome region maintenance 1 (Crm1)^89^ or Nxf2 adaptors to modulate target mRNA export^90^.

In summary, writer and eraser proteins ensure the timeliness and precision of gene expression through the dynamic regulation of RNA nuclear export.

### 3.3 Regulation of mRNA translation

Writer and eraser proteins precisely regulate mRNA translation efficiency through dynamic m^6^A modifications, maintaining protein synthesis homeostasis in cells. This regulation is crucial for normal physiological processes and also plays an important role in disease pathogenesis.

Following nuclear export, mRNA translation efficiency is modulated by m^6^A modifications regulated by writers (e.g., Mettl3 and Mettl16) and erasers (e.g., Fto). Mettl3 enhances the interaction between poly(A)-binding protein cytoplasmic 1 (Pabpc1)^91^ and eukaryotic translation initiation factor (eIf)4g by binding with Pabpc1, stabilizing eIf4f complex assembly, promoting the connection between the mRNA 5'-cap structure and the 3'-tail, facilitating ribosome recycling from termination sites back to the 5' end for subsequent translation rounds, ultimately boosting translation efficiency^92^. Mettl16 interacts with translation initiation factors (eIf3a, eIf3b) and rRNA to promote the assembly of the 43S pre-initiation complex and drive 80S initiation complex formation, ensuring that translation smoothly enters the elongation phase and thereby enhancing protein synthesis^7^. However, Fto diminishes the promotional effect of reader proteins (e.g., Ythdc1) on translation initiation by erasing m^6^A modifications from mRNA, reducing the loading of the 43S complex on mRNA and hindering the efficient assembly of the 80S complex^66^. This demethylation process reduces mRNA translational efficiency, thus forming a dynamic balance between the accuracy and speed of protein synthesis.

### 3.4 Regulation of mRNA decay

Decay, the final step in mRNA metabolism in which mRNAs become unstable and are degraded, is characterized by a precise mechanism involving writer and eraser proteins. Writer proteins (e.g., Mettl3, Mettl16) catalyze m^6^A deposition in mRNA subtelomeric regions, enhancing reader protein binding and shielding mRNA from RNase-mediated degradation to extend half-life. Stable m^6^A modifications further promote R-loop formation, supporting homologous recombination and telomere stability. Loss of Mettl3 and Mettl16 diminishes these m^6^A marks, accelerating mRNA decay and compromising telomere integrity and cellular function^93^.

Eraser proteins (e.g., Fto, Alkbh5) modulate mRNA stability by removing m^6^A modifications, influencing cancer progression and chemoresistance. Fto demethylates salt-inducible kinase 2 (Sik2) mRNA, reducing its binding to Igf2bp2 and promoting degradation, which inhibits autophagy and facilitates clear cell renal cell carcinoma proliferation and metastasis^94^. Alkbh5-mediated demethylation of Foxo1 mRNA enhances its stability, upregulating superoxide dismutase (Sod2) to lower Ros levels, thereby maintaining cancer stem cell traitsand conferring chemoresistance in triple-negative breast cancer; Alkbh5 depletion sensitizes cells to doxorubicin by decreasing Foxo1/Sod2 expression^95^. Dynamic m^6^A modification regulates RNA stability, tumorigenesis, and drug response, with m^6^A-binding proteins and demethylases directly linked to tumor cell growth and metastasis^96^, ^97^. Further studies reveal that m^6^A modification dynamic during ovarian development and aging are closely related to RNA stability and chromatin state^98^. This suggests a complex role for m^6^A modifications in modulating DNA epigenetics and cellular function. Therefore, the fine-tuned addition and removal of m^6^A modifications by writer and eraser proteins affect mRNA stability while also playing pivatol roles in cellular biological functions and tumor progression.

The coordinated actions of writer, eraser, and reader proteins are essential for dynamic m^6^A modification of RNA, governing processes ranging from RNA stability and splicing to nuclear export and translational efficiency. Dysregulation of these proteins to diverse diseases, emphasizing the importance of deciphering their functional networks. Further investigations into their functions in physiological and pathological contexts will deepen our understanding of RNA biology and inform novel therapeutic strategies for associated disorders.

## 4. Molecular mechanisms of methyltransferases and demethylases in the liver diseases

In recent years, the investigation of m^6^A RNA methylation has gained increasing attention in the field of liver diseases. Substantial evidence demonstrates that aberrant m^6^A modification is intimately associated with the pathogenesis and progression of various hepatic disorders, including ALI, NAFLD, liver cirrhosis, alcoholic liver disease (ALD), viral hepatitis, HF, and HCC. Elucidating the regulatory mechanisms of m^6^A modification will not only advance our understanding of disease pathogenesis but also potentially novel therapeutic targets for clinical intervention.

### 4.1 m^6^A modification in acute liver injury

The m^6^A modification plays a pivotal role in ALI, illustrating its dynamic regulation of liver diseases. Mettl3 employs m^6^A modification to regulate the expression of nuclear factor erythroid 2-related factor 2 (Nrf2), its downstream genes, and the lncRNA metastasis-associated lung adenocarcinoma transcript 1 (Malat1). Mettl3 deficiency aggravates oxidative stress and hepatocyte damage, while pregnane X receptor (Pxr) activation upregulates Fto, reduces m^6^A modification of Malat1, restores Nrf2 activity, and enhances antioxidant capacity. These findings identify Mettl3, Fto, and Pxr as critical therapeutic targets for liver injury prevention and treatment^99^. Mettl14 exerts hepatoprotective effects in endoplasmic reticulum (ER) stress-induced ALI by suppressing the pro-apoptotic factor C/EBP homologous protein (Chop). Under ER stress conditions, inhibition of the HMG-CoA Reductase Degradation 1 (Hrd1) ubiquitination pathway stabilizes Mettl14 expression, thereby reducing hepatocyte apoptosis. Notably, while Mettl14-deficient mice develop severe liver injury under stress conditions, genetic ablation of Chop (Ddit3) reverses this phenotype, underscoring the importance of the Mettl14-Chop axis in ER stress response^100^. Mettl3 promotes gluconeogenesis via stabilizing Phosphoenolpyruvate Carboxykinase 1 (Pck1) mRNA, reducing lactate accumulation and improving liver function. Both Pck1 and Mettl3 knockout exacerbates ischemia/reperfusion injury, emphasizing the protective role of the Mettl3/m^6^A-Pck1 pathway^101^. Mettl3 deficiency in ALI upregulates sphingomyelinase Smpd3, causing ceramide accumulation and mitochondrial/ER stress-induced apoptosis. Targeting the Mettl3-sphingolipid metabolism axis via Smpd3 inhibition or sphingomyelin synthase 1 (Sgms1) upregulation alleviates liver injury^102^. In acetaminophen-induced ALI, Wtap collaborates with Mettl3 and Mettl14 to enhance m^6^A-modified antioxidant and anti-apoptotic genes expression, maintaining metabolic homeostasis and inhibiting Jnk hyperactivation. Wtap downregulation exacerbates hepatocyte injury, suggesting Wtap complex activation as a therapeutic strategy^103^. It is worth mentioning that Fto emerges as a critical regulator in age-related ALI, where its reduced expression enhances m^6^A modification of Acsl4 (acyl-CoA synthetase long-chain family member 4) and Tfrc (transferrin receptor 1), promoting ferroptosis and exacerbating lipid peroxidation and Ros accumulation. In ischemia-reperfusion models, Fto overexpression suppresses ferroptosis related molecules and mitigating tissue damage. Therapeutically, enhancing Fto activity via nicotinamide mononucleotide (NMN) represents a promising strategy to alleviate liver transplantation injury in elderly individuals^104^.

While writers and erasers play crucial roles in ALI pathogenesis, the mechanisms of action of proteins such as Mettl3, Mettl14, and Fto in hepatocyte stress response, repair, and regeneration require elucidation. Specifically, the signaling pathway linking Pxr activation to Fto upregulates and affects the m^6^A modification of Malat1, and the upstream and downstream regulatory factors of the Mettl3 sphingolipid metabolism pathway in liver repair, require in-depth study. Exploration of these areas may guide the direction of future research on ALI.

### 4.2 m^6^A modification in viral hepatitis

The m^6^A modification plays a significant role in the interaction between chronic hepatitis B (CHB) and Covid-19, revealing its dynamic regulatory role in liver diseases. Clinical observations indicates that patients with CHB have an increased risk of hospitalization after contracting Covid-19. Furthermore, the immune disorders and liver injury induced by Covid-19 are exacerbated under abnormal m^6^A regulation^105^. Notably, Mettl3 enhances antiviral genes expression to activate the immune response; however, this can lead to uncontrolled inflammation^106^. Rbm15 contributes to Covid-19-associated hepatitis by binding SARS-CoV-2 RNA to modulate viral replication while simultaneously influencing host immune responses, particularly through regulation of key cytokines (Il-6, Tnf-α) and subsequent inflammatory cascades^107^. By contrast, Fto weakens RNA stability and interferes with antiviral immunity. These findings suggest that intervention strategies targeting m^6^A regulation could help to balance the antiviral and anti-inflammatory responses.

In hepatitis B virus (HBV)-related acute-on-chronic liver failure (ACLF), HBV infection upregulates Mettl3 and the miRNA miR-146a-5p, increases the m^6^A modification level, and exacerbates apoptosis, inflammation, and viral replication. Inhibiting Mettl3 can reduce hepatocyte damage and inhibit the maturation of miR-146a-5p to improve liver injury, highlighting Mettl3 as a key therapeutic target for ACLF^108^. Equally noteworthy is that, in HBV-induced acute liver failure (ALF) features dysregulated Mettl3, Igf2bp2, and Igf2bp3 expression that promotes immune cell infiltration (e.g. CD8^+^T cells and T helper 17 cells), with consequent Th17/Treg imbalance worsening hepatic injury. This has spurred development of m^6^A-based diagnostic models for early ALF detection and immunotherapeutic approaches^109^. The HBV X protein (HBx) upregulates Rbm15 to enhance m^6^A modification of circRNA Fam210a, accelerating its decay while activating Ybx1 transcriptional activity to drive hepatocarcinogenesis^110^. Additionally, HBV stimulates Kiaa1429-mediated m^6^A modification to upregulate Ccr9, which stabilizes drug transporters ATP-binding cassette subfamily B member 1 (Abcb1) and subfamily C member 1 (Abcc1) expression, fostering HCC chemoresistance and poor prognosis^111^. HBX further engage the m^6^A complex (Mettl3/Mettl14), recruiting it to viral cDNA to enhance transcript modification. This regulatory mechanism stabilizes viral transcription while reducing the expression of the HBX protein, forming a negative feedback loop to maintain CHB infection^112^.

Hepatitis C virus (HCV) exploits the m^6^A machinery (Wtap-Mettl3/14) to promote its life cycle and immune evasion. Wtap directs m^6^A complex positioning on HCV RNA, preventing retinoic acid-inducible gene I (Rigi) detection while boosting virion production^113^, ^114^.

The m^6^A modification has a negative regulatory effect on the hepatitis D virus (HDV) life cycle. Research shows that Mettl3/Mettl14-mediated m^6^A modification exerts negative regulation on HDV by reducing genomic RNA and delta antigen levels, yet paradoxically increasing extracellular genome accumulation. This modification further impedes virion assembly through Ythdc1-mediated interference with HDV genome-delta antigen interactions^115^. The demethylase Alkbh5 is upregulated under hypoxic conditions, reducing the m^6^A modification of HBV RNA to prolong its stability and promote viral replication^116^. Alkbh5 also enhances the stem cell-like properties and immune evasion of HCC by stabilizing Snai2 transcripts^117^. Meanwhile, alterations in Fto expression during HIV/HCV co-infection have been closely related to metabolic disorders, insulin resistance, and patient treatment response^118^. These two demethylases play crucial roles in the progression of viral infections by modulating viral RNA stability and host transcriptional responses, reducing the recognition of viral RNA by sensors such as Rigi, and enhancing the immune evasion ability of the virus^119^.

Although it is known that m^6^A modification affects the stability and translation efficiency of viral RNA, other molecular aspects of its regulation in hepatitis virus infection require further research. Specifically, the mechanisms by which hepatitis viruses use the host cell's m^6^A modification system to evade the recognition and clearance of the immune system, as well as how the host cell resists virus infection by regulating m^6^A modification, remain unclear.

### 4.3 m^6^A modification in NAFLD

**Figure 2:**
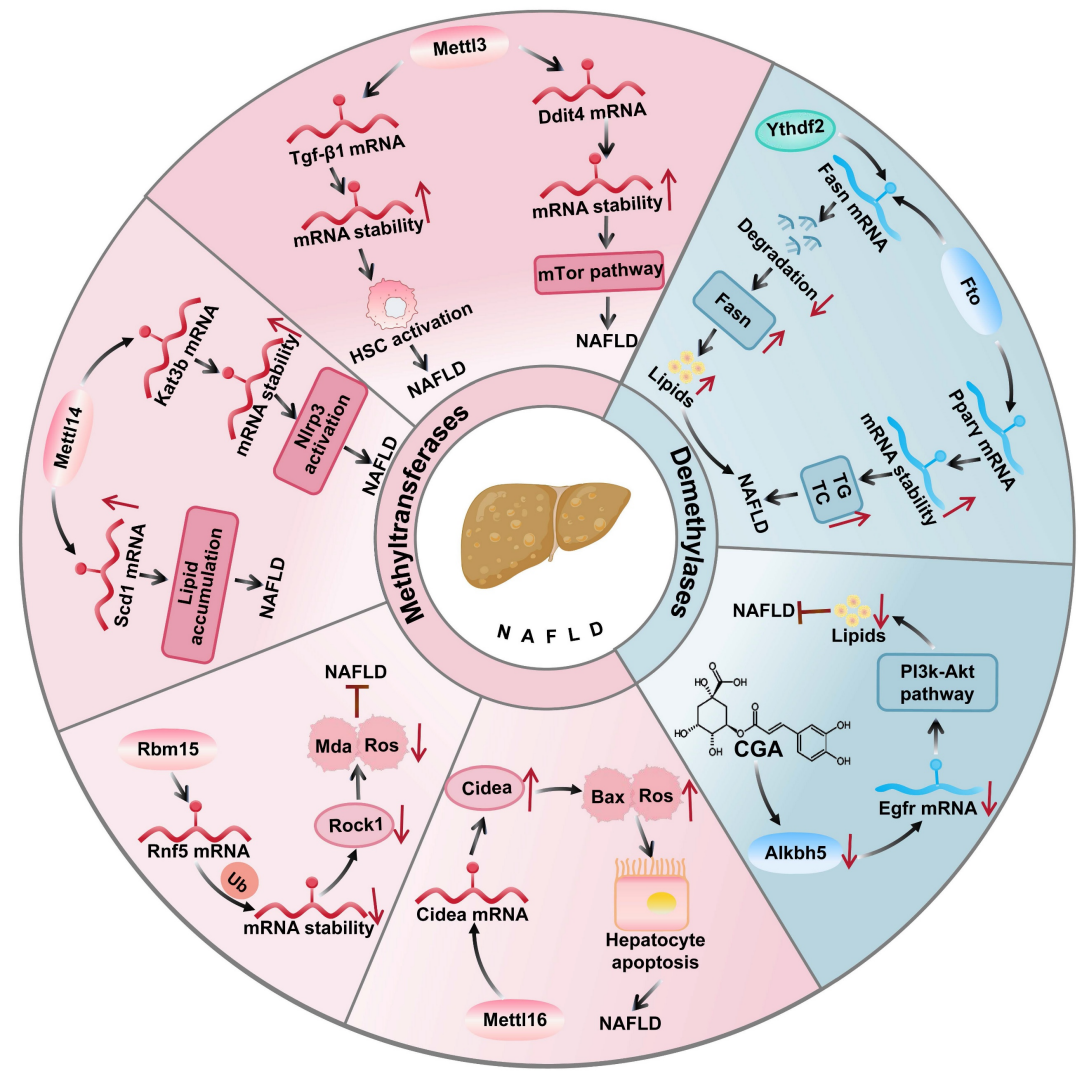
** The role of m^6^A writer and eraser proteins in NAFLD.** In NAFLD, Mettl3 promotes disease progression by stabilizing Ddit4 mRNA to activate the mTor pathway and enhancing Tgf-β1 mRNA to drive liver fibrosis, while Mettl14 accelerates NAFLD by increasing Kat3b-mediated Nlrp3 expression. Conversely, Mettl3 inhibits hepatic lipid metabolism through Cd36 and Ccl2 regulation, Rbm15 reduces oxidative stress by destabilizing Rnf5 mRNA, and Fto attenuates NAFLD progression by suppressing Srebf1 mediated lipogenesis.

The role of m^6^A modification in NAFLD pathogenesis and therapeutic targets m^6^A modification crucially regulates NAFLD progression by modulating lipid metabolism, inflammatory responses, and cellular processes through m^6^A-related enzymes and target gene stability (Figure 2).

The main writer Mettl14 promotes inflammation by enhancing the stability of Nlrp3 inflammasome mRNA^120^, while arsenite increases Nlrp3 m^6^A modification exacerbating NAFLD^121^. Lipopolysaccharide activates NF-κB p65 to transcribe Mettl3 and Mettl14, boosting Tgf-β1 mRNA m^6^A modification in the 5'-UTR for cap-independent translation and NAFLD progression^22^. Additionally m^6^A modification significantly influences in NAFLD and obesity, especially in the function of Mettl3 in myeloid cells^122^. Mettl3 in myeloid cells regulates Ddit4 mRNA stability via m^6^A to inhibit mTor/NF-κB signaling, counteracting NAFLD and obesity^123^.

The autophagy mechanisms of m^6^A modification in NAFLD cannot be ignored. In NAFLD mouse models and free fatty acid-treated hepatocytes, elevated m^6^A modification levels correlate with Mettl3 upregulation^124^. In autophagic mechanisms, Mettl3 upregulation in NAFLD models modifies Rubicon mRNA, promoting its expression via Ythdf1, which blocks autophagosome-lysosome fusion and lipid clearance^125^. Mettl3-mediated Cyp2b6 m^6^A modification increases its expression, inhibiting insulin receptor substrate phosphorylation and exacerbating insulin resistance^126^. Further studies found that the overexpression of Mettl14 and Mettl3 promotes fatty acid synthesis and lipid accumulation by stabilizing the mRNAs of ATP citrate lyase (Acly) and stearoyl-CoA desaturase 1 (Scd1) and accelerating NAFLD to HCC^127^, while adipose tissue Mettl3 and Mettl14 modify Adrb2/3, adipose triglyceride lipase (Atgl), and comparative gene identification 58 (Cgi58) transcripts to impair β-adrenergic signaling, reducing lipolysis and worsening obesity/NAFLD^128^. Mettl16 promotes NAFLD via m^6^A mediated cell death inducing DFFA-like effector A (Cidea) upregulation^129^, whereas Rbm15 reduces NAFLD by m^6^A-methylating ring finger protein 5 (Rnf5) to ubiquitinate and degrade Rock1^130^.

**Figure 3:**
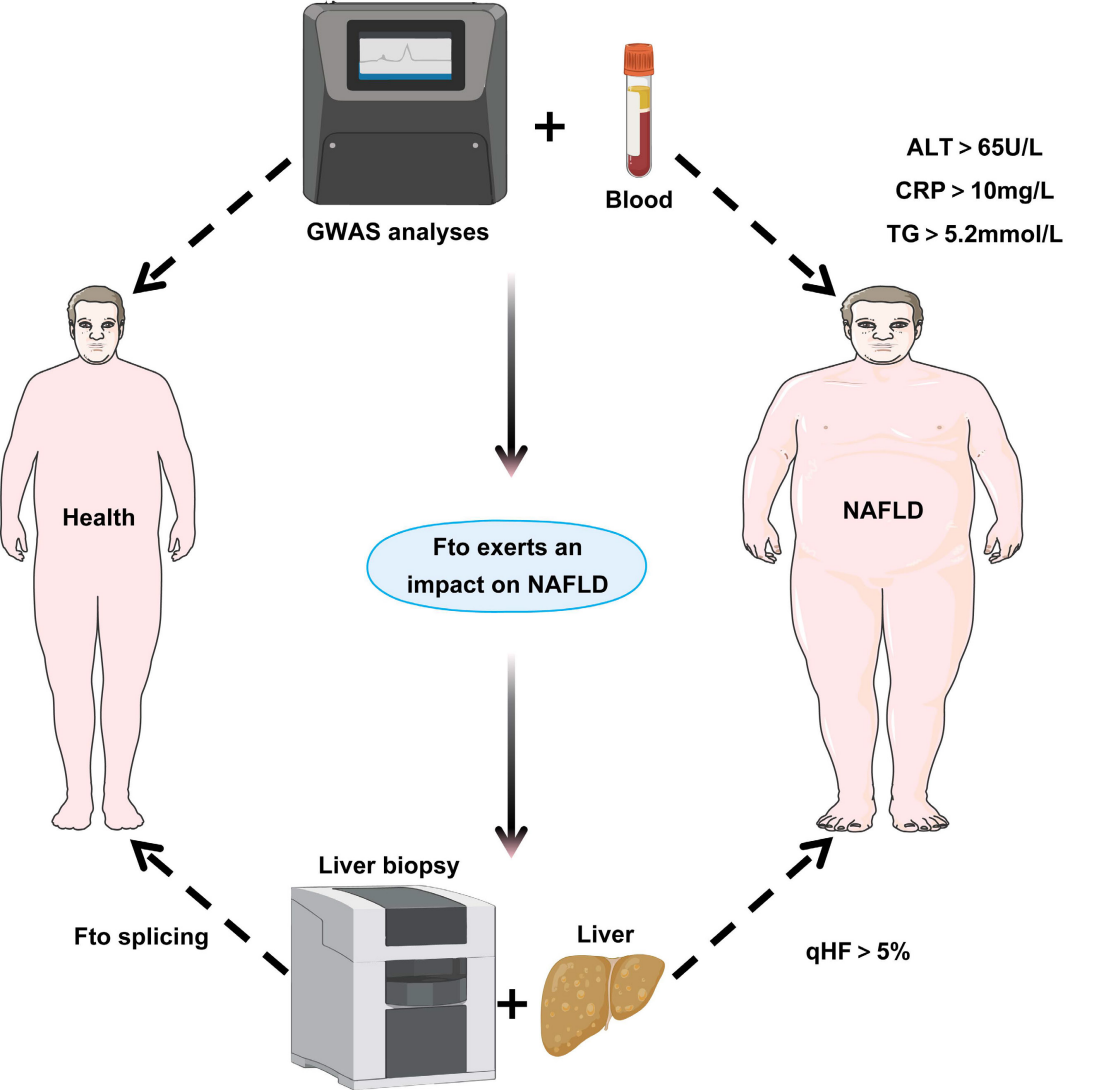
** Clinical relevance of Fto in NAFLD.** By collecting blood samples from healthy individuals and NAFLD patients, measuring key clinical parameters (ALT > 65 U/L, C-reactive protein (CRP) > 10 mg/L, Triglyceride (TG) > 5.2 mmol/L), and integrating GWAS data, this study combines hepatic biopsy for quantitative hepatic fat (qHF > 5%) assessment in NAFLD patients, followed by analysis of Fto splicing patterns in liver tissues to elucidate its impact on NAFLD development and progression.

In the treatment of NAFLD, the demethylases Fto and Alkbh5 are targeted for their regulation of metabolism and inflammatory responses via the removal of m^6^A modifications. Fto upregulation enhances lipogenic gene expression and promotes lipid accumulation^131^, while Alkbh5 affects proinflammatory genes^132^. Specifically, Fto catalyzes m^6^A demethylation altering the expression and splicing of lipid-related genes. As shown in (Figure 3), in the liver, Fto upregulation enhances sterol regulatory element binding protein 1c (Srebp1c) mediated lipogenesis which inhibiting fatty acid oxidation to exacerbate NAFLD^133^. By contrast, angiotensin-receptor blockers inhibit Fto to increase solute carrier family 7 member 11 (Slc7a11) m^6^A modification and suppress ferroptosis. Furthermore, chlorogenic acid (Cga) promotes autophagy and reduces lipid deposition by inhibiting the m^6^A demethylase activity of Alkbh5, revealing the potential application of m^6^A modification in the treatment of NAFLD^134^.

### 4.4 m^6^A modification in ALD

The progression of ALD encompasses multiple stages, from alcoholic fatty liver to cirrhosis and even hepatocellular carcinoma. In recent years, m^6^A, one of the most common types of mRNA modification, has been proven to play multifaceted regulatory roles in ALD development and progression. First, m^6^A modification affects the formation of alcoholic fatty liver by regulating the expression of genes related to lipid metabolism. Research shows that elevated m^6^A levels enhance the translation of lipogenic genes, promoting lipid synthesis and hepatic steatosis, while reduced the expression of the demethylase Fto may further exacerbate lipid accumulation^135^. Second, in alcoholic hepatitis, m^6^A modification exhibits a dual regulatory role in the liver inflammatory response. The m^6^A reader protein Ythdf2 promotes the degradation of inflammatory factors, thus alleviating inflammation^136^. However, stimulation with alcohol may change the m^6^A level, upregulating proinflammatory factors and exacerbating liver injury. Studies have shown that alcohol intake can trigger Kupffer cell pyroptosis and increase the release of proinflammatory factors such as Il-1β. Through regulation by the RNA-modifying enzyme Mettl3, m^6^A modification influences the inflammatory response and pyroptosis in Kupffer cells. Specifically, inhibiting Mettl3 was shown to relieve the inflammatory cascade reaction caused by Kupffer cell pyroptosis, thus reducing pathological damage in alcoholic steatohepatitis^137^. Fto, by reducing the m^6^A modification level of the Il-17a receptor gene Il-17ra, increasing its protein expression, thereby exacerbating the inflammatory response in the liver. Fto upregulation elevates Il-17-related inflammatory factors, whereas Fto inhibition enhances Il-17ra m^6^A modification, lowering its protein levels and attenuating inflammation^24^. In addition, at the stage of alcoholic HF, m^6^A modification affects fibrosis-related genes. For instance, m^6^A modification may affect the stability of key genes in the Tgf-β signaling pathway, thus promoting or inhibiting fibrosis^138^. Finally, during the progression of ALD to liver cancer, m^6^A modification influences the expression of oncogenes and tumor suppressor genes, with abnormalities promoting the proliferation and invasion of liver cancer cells, driving disease progression. Therefore, m^6^A modification affects different stages of ALD through multiple pathways, having a profound impact on the disease process.

In summary, m^6^A modification exerts multilevel regulatory effects on ALD pathogenesis, impacting key processes including lipid metabolism, inflammation, fibrosis, and carcinogenesis. Further investigation into the molecular mechanisms of m6A modification and its stage-specific roles in ALD may provide novel insights into pathogenesis machanisms. Such researchs could also uncover potential diagnostic biomarkers and therapeutic targets, paving the way for personalized treatment strategies.

### 4.5 m^6^A modification in HF

**Figure 4:**
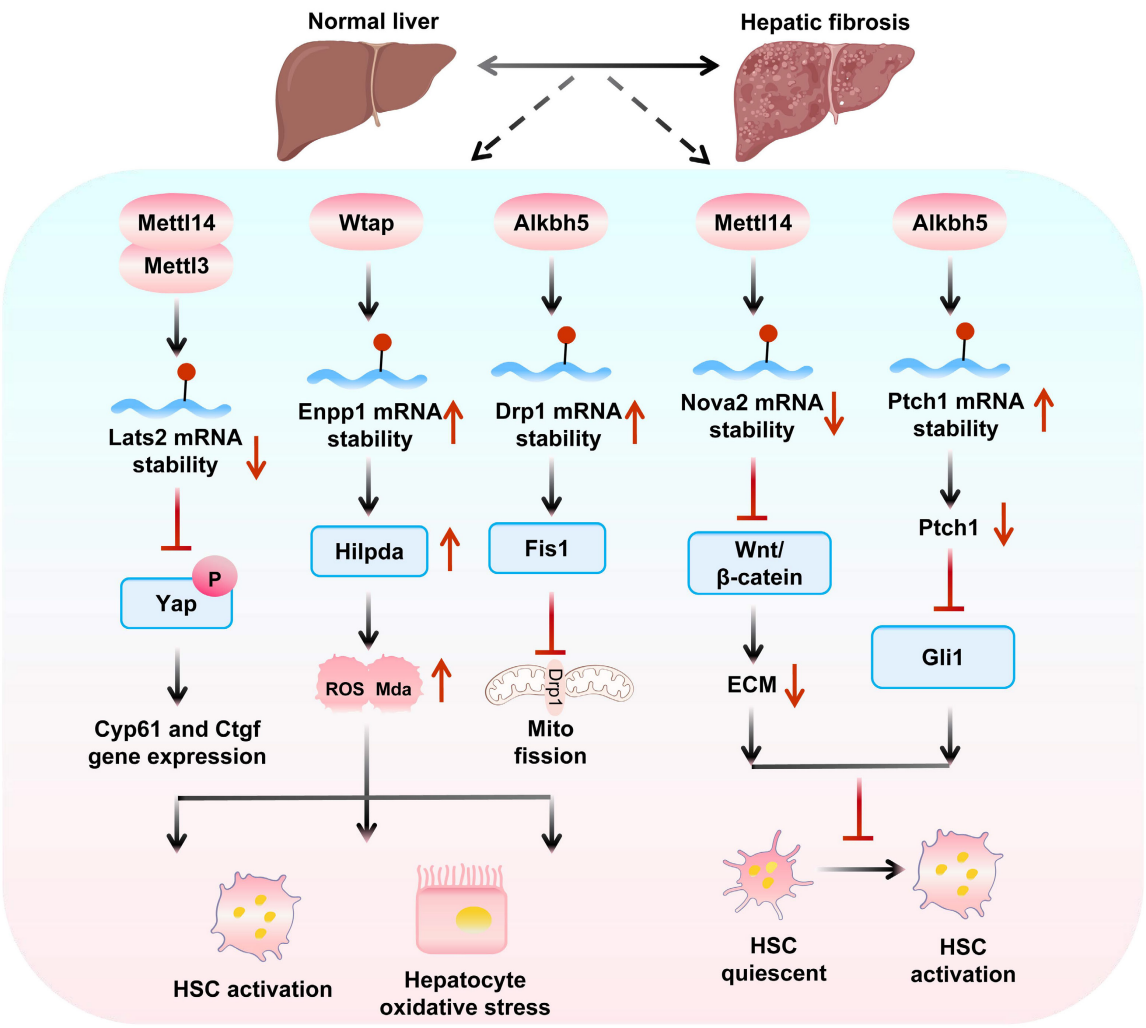
** Mechanisms of m^6^A “writers” and “eraser” in hepatic fibrosis.** m^6^A writers (Mettl14/Mettl3/Wtap) promote fibrosis by destabilizing Lats2 to activate Yap-driven HSC activation and stabilizing Enpp1 to upregulate Hilpda-mediated oxidative stress. Conversely, Alkbh5 plays a dual regulatory role in hepatic fibrosis: while enhancing Drp1 expression to promote mitochondrial fission and oxidative stress, it simultaneously suppresses fibrosis by destabilizing Nova2 to inhibit Wnt/β-catenin signaling and stabilizing Ptch1 to block Gli1 activation, thereby comprehensively modulating HSC activation.

In HF and related diseases, m^6^A modification regulates key gene expression, hepatic stellate cell (HSC) activation, and cell-cell interactions, highlighting its potential as a therapeutic target and providing new research directions (Figure 4).

The role of m^6^A modification-related enzymes in hepatic fibrosis and therapeutic implications Mettl14 exerts dual effects on HF progression via reader protein-dependent m^6^A mechanisms. On one hand, the Mettl14 and Ythdf1 axis reduces glutaminase 2 (Gls2) translation, creating an oxidative stress microenvironment that recruits Cx3cr1+/Ccr2+ monocytes. Crucially, Cx3cr1 activates the NF-κB pathway via Myd88, leading to transcriptional upregulation of profibrotic factors like S100a4, which drives HSC activation and fibrosis^139^. On the other hand, Mettl14 decreases m^6^A modification of Nova2 mRNA, enabling Ythdf2-mediated degradation and inhibit Nova2 activity^140^, Conditional knockout of Mettl3, decreases m^6^A modification of Lats2 mRNA, thereby inhibiting the nuclear entry of Yap, downregulates profibrotic genes and causing HSCs to switch from an activated state to a quiescent state^141^, blocking HF progression.

Sodium arsenite (NaAsO_2_) enhances m^6^A modification, promoting Mettl14 and Igf2bp2-mediated stability of Tgf-β1 mRNA; limiting this modification prevents NaAsO_2_-induced HSC activation^142^. Ectonucleotide pyrophosphatase and phosphodiesterase 1 (Enpp1), upregulated via Wtap-mediated m^6^A modification and Ythdf1-dependent translation^143^, exacerbates fibrosis by promoting HSC lipid oxidation and proliferation. Conversely, N-acetyl-serine-aspartic acid-lysine-proline (AcSDKP) inhibits Hedgehog signaling by stabilizing patched 1 (Ptch1) mRNA via Wtap downregulation^144^.

In the Fto/Unc51 like autophagy activating kinase 1 (Ulk1) axis, Fto promotes Ulk1-mediated autophagy and HSC activation, whereas Ythdc2-mediated Ulk1 regulation inhibits HF^145^. During ferroptosis, m^6^A modification stabilizes Becn1 mRNA to activate autophagy^94^, a process enhanced by Ythdf1 and downregulated Fto. Dihydroartemisinin (DHA) inhibits HSC activation via Fto-upregulated autophagy and ferroptosis^146^.

Alkbh5 exhibits context-dependent roles in HF. In Tgf-β-1-stimulated HSCs, Alkbh5 overexpression reduces Snail1 mRNA stability and suppresses profibrotic markers^23^, while simultaneously activating Ptch1 to inhibit HSC activation^147^ and blocking Drp1 mRNA m^6^A modification to restrain mitochondrial fission-dependent HSC proliferation/migration^148^. Notably, reduced Alkbh5 expression exacerbates HF progression, positioning it as a protective factor in fibrotic microenvironments. Given that the decreased expression of Alkbh5 exacerbates HF, research on the regulation of its demethylation activity is expected to open up new directions for the treatment of HF.

### 4.6 m^6^A modification in liver cirrhosis

Liver cirrhosis, characterized by extensive replacement of liver parenchyma with fibrous tissue and a significant decline in liver function, represents the terminal stage of liver diseases. Emerging evidences have shown that m^6^A-modified mRNAs play an important role in the pathogenesis and progression of liver cirrhosis. As a dynamic and reversible mode of epigenetic RNA regulation, m^6^A modification influences the key pathological processes of liver cirrhosis via regulation of gene expression, RNA stability, translation efficiency, and other mechanisms. As one of the main drivers of liver cirrhosis, the Tgf-β signaling pathway stabilizes its downstream Smad genes through m^6^A modification, thereby amplifying fibrogenic signal. This accelerates the activation of HSCs and the accumulation of extracellular matrix, promoting the development of fibrosis^149^, ^150^. In cirrhotic tissues, elevated expression of m^6^A writer enzymes Mettl3 and Mettl14 enhances the translation efficiency of fibrogenic genes, aggravating the fibrotic process and promoting the progression of liver cirrhosis^151^. Conversely, low expression of the m^6^A demethylase Fto leads to increased m^6^A modification of profibrotic genes, stabilizing their mRNAs and promoting the accumulation of fibrosis^152^. In addition, reduced levels of the m^6^A-binding protein Ythdf2 hinders the degradation of fibrotic and proinflammatory genes, resulting in the continuous expression of these genes and exacerbating the inflammatory and fibrotic responses. The m^6^A-mediated stabilization of proinflammatory cytokines Il-6 and Il-1β also perpetuates chronic inflammation, further driving cirrhosis progression^153^. Through these multifaceted mechanisms that regulates fibrotic and inflammatory gene expression, m^6^A modification significantly contributes to cirrhosis development. As an important epigenetic regulatory mechanism, m^6^A modification machinery provide new potential targets for the treatment of liver cirrhosis. Future therapies targeting m^6^A modification may potentially slow disease progression, opening new avenues for more effective treatment strategies.

### 4.7 m^6^A modification in HCC

**Figure 5:**
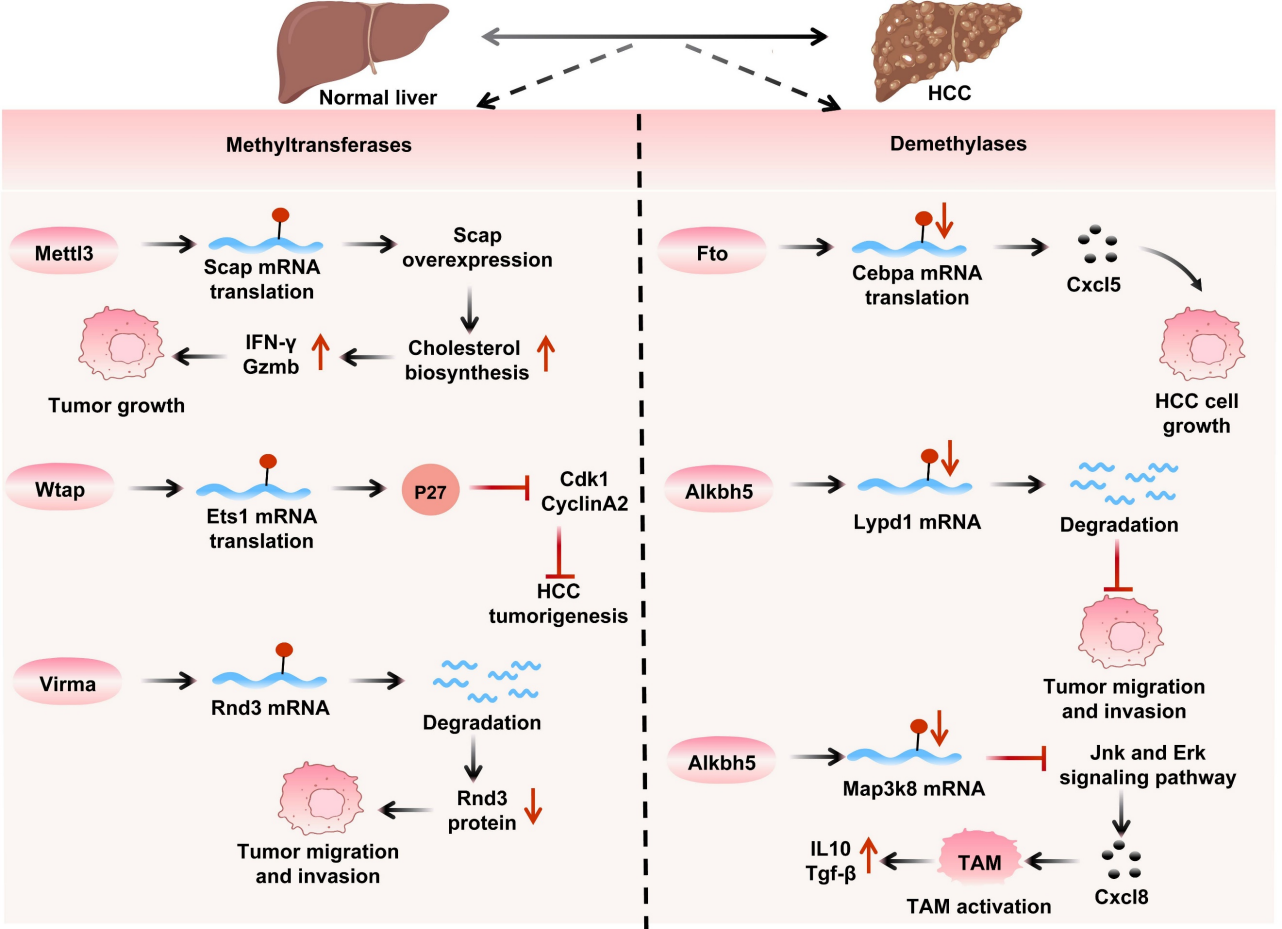
** The role of m^6^A writer and eraser proteins in HCC.** Methyltransferases: Mettl3 activates cholesterol synthesis via Scap mRNA translation to promote tumor growth; Wtap regulates P27 through Ets1 mRNA for HCC oncogenesis; Virma degrades Rnd3 mRNA to drive migration/invasion. Demethyltransferases: Fto generates Cxcl5 via Cebpa mRNA translation to facilitate HCC cell growth; Alkbh5 degrades Lypd1 mRNA to enhance migration, and activates tumor-associated macrophages (TAM) via Map3k8 mRNA, exacerbating migration/invasion through Jnk/Erk pathway and elevated Il10/Tgf-β, collectively demonstrating how m^6^A modifications influence HCC development and tumor microenvironment.

As illustrated in (Figure 5), research on HCC has shown that m^6^A modification significantly impacts tumor progression and treatment response. Mettl3-mediated m^6^A modification stimulates Egfr mRNA translation, leading to lenvatinib resistance^154^. While Mettl3 is significantly upregulated in HCC and associated with shorter survival, with its knockout inhibiting tumorigenicity and metastasis. Mettl3 mediates Socs2 degradation, modulates Snail's epithelial-mesenchymal transition, and promotes cell proliferation/lipid production via modifying Rdm1 and stabilizing Linc00958^155^, ^156^, forming a positive feedback loop with Stat3 by suppressing anti-tumor CD8^+^T cells through the Scap-cholesterol axis^157^, promoting Stat3 mRNA translation, and accelerating metastasis as Stat3 upregulates Wtap to enhance Mettl3's nuclear function^158^. Additionally, Mettl3 promotes Tug1 upregulation to regulate Pd-l1/Cd47 and affect immune escape^159^.

Mettl14-induced m^6^A modification stabilizes Usp48 and Sirt6, suppressing HCC glycolysis and malignancy^160^, reduces circORC5 expression to inhibit gastric cancer, and enhances circSTX6 in HCC^161^, while possibly downregulating Hnf3γ (which is negatively correlated with malignancy/survival) via m^6^A modification, with exogenous Hnf3γ promoting liver cancer cell differentiation and growth inhibition^162^.

Mettl16, upregulated in HCC and associated with poor prognosis, binds to lncRNA Rab11b-as1 inducing its m^6^A modification and degradation, promoting cell proliferation/migration. Targeting the Mettl16-eIf3a/b axis may represent a novel anti-cancer strategy^7^, ^163^. Wtap-mediated m^6^A modification stabilizes lnc-OXAR via Igf2bp2, leading to oxaliplatin resistance in NASH-related HCC^164^, promotes HCC progression via the HuR-est1-p21/p27 axis^165^ and upregulates the expression of autophagy-related 5 (Atg5), promoting its translation during ferroptosis. HBX-interacting protein (Hbxip) enhances cisplatin resistance in liver cancer cells by upregulating Wtap and its m^6^A modification of poly (Adp-ribose) polymerase (Parp1)^166^.

Regarding other related writers, Hakai interacts with Ajuba to enhance the proliferation of HCC cells^167^. HBX promotes the expression of Rbm15, increasing m^6^A levels on Fam210a mRNA and inducing its degradation, thereby improving the proliferation, stemness, and tumorigenicity of HCC cells. Kiaa1429 induces m^6^A methylation in the 3′-UTR of Gata Binding Protein 3 (Gata3) precursor mRNA, downregulating the expression of Gata3 and promoting the metastasis of HCC cells^168^.

As m^6^A demethylases, Fto (highly expressed in HCC) is stabilized by Fto-interacting transcript 1 (Fto-It1) to upregulate Glut1/Pkm2/C-myc for glycolysis and proliferation^126^. Meanwhile FB23 and FB23-2 inhibitors increase Erbb3/Tubb4a m^6^A levels to inhibit Akt-Mtor signaling^169^. Additionally, adenosylmethionine decarboxylase 1 (Amd1) upregulates Sry-box transcription factor 2 (Sox2), Kruppel-like factor 4 (Klf4), and Nanog through Fto-mediated mRNA demethylation, maintaining tumor stemness^170^.

Downregulation of Alkbh5 expression exerts a potential tumor and suppressive effect in HCC by inhibiting the transcription of Lypd1^171^. However, Alkbh5 has also been demonstrated to possess significant pro-tumorigenic functions: it enhances cancer stem cell properties and immune evasion by stabilizing Snai2^117^, and its target, Linc02551, similarly drives HCC growth and metastasis^172^. Intriguingly, in radiation-induced hepatic stellate cells (HSCs), Alkbh5 activation promotes chemokine secretion via the Tirap/NF-κB signaling pathway^109^, a mechanism potentially contributing to HCC radioresistance. This marked functional dichotomy observed across disease contexts highlights a critical unresolved question: how specific microenvironmental factors precisely regulate Alkbh5's substrate specificity and downstream signaling cascades. Future research elucidating the underlying mechanisms holds promise for developing context-specific therapeutic interventions targeting liver disease progression.

## 5. Investigation and advancement m^6^A of methylation-associated pharmaceuticals

In the treatment of liver diseases, novel chemical compounds influencing m^6^A modification have enabled m^6^A-based therapies, regulating activities of m^6^A-related enzymes. Fto inhibitors like Fb23 and Fb23-2 alter m^6^A levels of Erbb3 and signaling pathways, suppressing liver cancer cell proliferation and survival^173^-^175^, while Dac51 inhibits Fto-mediated glycolysis, showing enhanced efficacy when combined with anti-PD-L1 blockers^176^. STM2457, a Mettl3 inhibitor, reduces Egfr's m^6^A modification to increase HCC cells sensitivity to lenvatinib^177^, and UZH1A selectively binds to Mettl3 mRNA to exert antitumor effects in leukemia and osteosarcoma^178^. In NAFLD, STM2457 improves mitochondrial function and lipid oxidation^179^. ARBs inhibit Fto demethylation and promote Slc7a11 expression^180^, and Cga/MV1035 inhibit Alkbh5 to enhance autophagy^181^. In ALI, NMN enhances Fto activity to alleviate liver transplantation injury^130^.

Notably, traditional Chinese medicines show unique advantages: Resina Draconis extract induces HCC cell apoptosis by downregulating Mettl3^182^; Ling Gui Zhu Gan soup reduces liver fat degeneration by decreasing m^6^A methylation and Socs2 expression^183^; Gan Jiang Ling Zhu soup promotes Mettl14 and Ugt2a3 expression to alleviate NASH^184^; indole-3-lactic acid regulates Cyp8b1 via Fto/m^6^A/Ythdf2 to inhibit liver fat accumulation^185^; and cucurbitacin B covalently binds to Igf2bp1 to block m^6^A-modified mRNA recognition, offering new directions for liver cancer drug development^186^.

## 6. Therapeutic implications and future directions

Emerging evidence have demonstrated that RNA m^6^A modification plays a pivotal role in the pathogenesis and progression of liver diseases. Elucidating the molecular mechanisms linking m6A modification to liver disease progression and may reveal promising therapeutic targets for drug development. Notably, targeting m^6^A regulators not only enhances treatment efficacy by modulating the liver disease microenvironment but also effectively overcomes drug resistance in clinical therapy, demonstrating promising application prospects.

Despite significant advancements in this field, only a limited number of m^6^A-targeting modulators have demonstrated ideal therapeutic efficacy in clinical applications for liver diseases. This limitation arises from multiple factors, including the lack of precision in current modulators for m^6^A modification regulation, often leading to off-target effects. For example, the Mettl3 inhibitor STM2457 may interfere with other RNA-modifying enzymes, disrupting snRNA methylation and causing global transcriptomic dysregulation^177^. Moreover, targeted interventions can induce compensatory feedback mechanisms, such as the upregulation of Fto and Alkbh5 following Mettl3 suppression, potentially counteracting therapeutic benefits^187^. Beyond specificity challenges, a key obstacle lies in the predominant focus on modulator activity optimization while overlooking critical pharmacokinetic properties, including absorption, distribution, metabolism, excretion, and lipophilicity. To address this, future research should prioritize the development of advanced delivery systems, such as TAT peptide-functionalized PLGA nanoparticles^156^ or folate-modified exosome-liposome hybrid nanocarriers to enhance^188^ tissue-specific drug delivery. Furthermore, the tissue-specific nature of m^6^A regulation introduces additional complexity. While hepatocyte specific Mettl3 upregulation exacerbates lipid accumulation in NAFLD^123^, in contrast, it exerts anti-inflammatory effects in myeloid cells by stabilizing Ddit4 mRNA^125^. This stark functional dichotomy underscores the need for highly selective therapeutic strategies to avoid unintended systemic effects.

To address these challenges, future investigations should focus on three pivotal research directions: (1) Deciphering cell type-specific m^6^A epitranscriptomic landscapes in hepatic microenvironments through single-cell sequencing approaches; (2) Establishing physiologically relevant organoid models that faithfully recapitulate liver microenvironments for comprehensive pharmacodynamic evaluation of m^6^A modulators; (3) Engineering tissue-specific delivery platforms to enable precision therapeutic modulation. These strategic advancements will substantially facilitate the translation of m^6^A-targeted therapeutics from fundamental research to clinical implementation.

## Figures and Tables

**Table 1:** The role of m^6^A methyltransferases and demethyltransferases in liver diseases

Type	Diseases	m^6^A regulators	Target genes	Functions
Core catalytic writer	ALI	Mettl14↑	Ccl2, Ccl5↓	Suppress inflammatory response and ameliorate hepatic damage
Core catalytic writer	ALI	Mettl3↑	Pck1↑	Promote gluconeogenesis and reduces lactate accumulation
Core catalytic writer	NAFLD	Mettl3↓	Cd36↓	Drive NAFLD to NASH progression
Core catalytic writer	NAFLD	Mettl3↑	Rubicon↑	Suppress autophagy impairs lipid droplet clearance
Core catalytic writer	NAFLD	Mettl14↓	Nlrp3↑	Promote hepatic inflammation and exacerbates liver injury
Core catalytic writer	NAFLD	Mettl16↑	Cidea↑	Promote hepatic lipid accumulation and metabolic dysregulation
Accessory factor	NAFLD	Rbm15↑	Rock1↓	Suppress lipid synthesis and inflammation
Core catalytic writer	HF	Wtap↓	Ptch1↑	Suppress aberrant fibrotic progression
Core catalytic writer	HCC	Mettl3↑	State3↑	Enhance nuclear translocation and evasion of tumor cells
Core catalytic writer	HCC	Mettl14↑	Usp48↑	Reduce glycolytic activity and malignancy in HCC
Accessory factor	HCC	Kiaa1429↑	Gata3↓	Enhance the migratory and invasive capacities of HCC
Core catalytic writer	HCC	Mettl3↑	Egfr↑	Lenvatinib treatment resistance
Accessory factor	HCC	Cbll1/Hakai↓	Ajuba↓	Promote the growth of HCC cells and tumors
Accessory factor	—	Zcchc4	—	Site specific m^6^A methylation of 28S rRNA
Accessory factor	—	Zc3h13	—	Interact with Wtap and bind to Rbm15 and Rbm15b
Accessory factor	—	Znf217	—	Mediate m^6^A RNA methylation through targeted DNA binding
m^6^A eraser	NAFLD	Fto↑	Pparγ↑	Suppress of hepatic steatosis
m^6^A eraser	NAFLD	Alkbh5↑	Linc01468	Promote hepatic inflammation and exacerbates liver injury
m^6^A eraser	ALD	Fto↑	Il-17ra↑	Recruit immune cells and exacerbate hepatic inflammation
m^6^A eraser	HF	Fto↑	Becn1↓	Suppress of autophagy mitigates ferroptosis
m^6^A eraser	HCC	Fto↑	Sox2, Klf4↑	Maintain of cancer stem cell properties
m^6^A eraser	HCC	Alkbh5↑	Tirap↑	Reduce the radiosensitivity of hepatocellular carcinoma cells

## Abbreviations

Mettl1: Methyltransferase-like1
Mettl3: Methyltransferase-like3
Mettl5: Methyltransferase-like5
Mettl10: Methyltransferase-like10
Mettl14: Methyltransferase-like14
Mettl16: Methyltransferase-like16
Virma/Kiaa1429: Vir-like m6A methyltransferase associated
Wtap: Wilms tumor 1- associated protein
Zcchc4: Zinc Finger CCHC-type Containing 4
Znf217: Zinc Finger Protein 217
Hsp90: Heat Shock Protein 90
Stk33: Serine/Threonine Kinase 33
Clip1: Cytoplasmic Linker Protein 1
Trmt112: tRNA methyltransferase 112
Rbm15/Rbm15b: RNA binding motif protein 15/15B
Zfp217: Zinc Finger Protein 217
Zc3h13: Zinc Finger CCCH-type Containing 13
Pcif1: Phosphorylated CTD-interacting factor 1
Cbll1/Hakai: E3 ubiquitin protein ligase Hakai
Fto: Fat mass and obesity-associated
Alkbh5: AlkB homologue 5
ALI: Acute liver injury
NAFLD: non-alcoholic fatty liver disease
HF: liver fibrosis
HCC: hepatocellular carcinoma
Pck1: phosphoenolpyruvate carboxykinase 1
Fmrp: fragile X mental retardation protein
Crm1: chromosome region maintenance 1
